# Atherosclerosis and Inflammation: Are the Rules of the Game Changing with Biological Therapies?

**DOI:** 10.2147/JIR.S531345

**Published:** 2025-07-24

**Authors:** Rıdvan Bora, Ahmet Turhan Kılıç, Burak Toprak

**Affiliations:** 1 Department of Cardiology, Tarsus State Hospital, Mersin, Turkey; 2 Department of Cardiovascular Surgery, Mersin City Education and Research Hospital, Mersin, Turkey

**Keywords:** atherosclerosis, inflammation, biological therapy, IL-1β inhibitors, IL-6 inhibitors, TNF-α inhibitors, cardiovascular disease

## Abstract

**Background:**

Atherosclerosis is a chronic, progressive vascular disease characterized not only by lipid accumulation but also by sustained inflammation. Recent evidence has highlighted the critical role of immune pathways and pro-inflammatory cytokines in plaque formation, progression, and destabilization.

**Aim:**

This narrative review aims to summarize current knowledge regarding the role of inflammation in atherosclerosis and evaluate the therapeutic potential of biologic agents targeting specific inflammatory pathways.

**Methods:**

A comprehensive literature search was conducted using keywords such as “atherosclerosis”, “inflammation”, “biologic agents”, and “cytokine inhibitors”. Recent clinical trials and relevant experimental studies were analyzed and synthesized narratively.

**Results:**

Biologic agents targeting IL-1β, IL-6, IL-17A, and CD20+ B cells have shown promising results in reducing inflammation and improving cardiovascular outcomes in high-risk patients. Agents such as canakinumab, anakinra, tocilizumab, rituximab, secukinumab, and alirocumab demonstrate varying degrees of efficacy depending on the targeted pathway. However, concerns remain regarding immunosuppression, lipid alterations, cost, and long-term safety.

**Conclusion:**

Biologic agents offer a novel, mechanism-based therapeutic approach in managing atherosclerosis. While current data support their benefit in select patient groups, further research is needed to clarify long-term outcomes, safety, and real-world applicability. Personalized treatment strategies based on inflammatory profiles may enhance clinical benefit.

## Key Points

**What is known about the topic?**

- Atherosclerosis is a chronic vascular disease caused by the combination of lipid accumulation and inflammation within the arterial wall.
- Inflammation plays a central role in all stages of atherosclerosis, from its initiation to progression.
- Pro-inflammatory cytokines such as IL-1β, IL-6, and TNF-α trigger inflammatory processes, leading to the destabilization and rupture of atherosclerotic plaques.
- Biologic agents suppress inflammation by targeting these inflammatory pathways, thereby reducing the risk of cardiovascular events.
- Studies such as CANTOS, ASSAIL-MI, and VCUART3 have demonstrated that pathway-specific inflammation suppression can lower cardiovascular events and improve myocardial outcomes.

**What does this study add?**

- This article presents a narrative overview of the roles, potential benefits, and limitations of inflammation-targeted biological therapies in atherosclerosis.
- While the CANTOS study showed that the IL-1β inhibitor canakinumab reduced cardiovascular events, the VCUART3 trial demonstrated that the IL-1 receptor antagonist anakinra decreased systemic inflammation and heart failure incidence in STEMI patients.
- The ASSAIL-MI and NSTEMI trials revealed that tocilizumab, an IL-6 receptor blocker, reduced myocardial injury and inflammatory biomarkers in acute coronary syndromes.
- The RITA-MI trial showed that rituximab safely depleted CD20+ B cells in STEMI patients, opening avenues for adaptive immune modulation.
- This study emphasizes that biologic agents targeting specific inflammatory pathways offer therapeutic potential when tailored to the individual patient’s risk profile and inflammatory burden.
- Furthermore, it suggests that personalized treatment strategies, supported by biomarkers and emerging agents such as secukinumab and alirocumab, may enhance future atherosclerosis management.

## Introduction

Atherosclerosis is a chronic, progressive vascular disease characterized by the accumulation of lipids, inflammatory cells, and fibrous tissue within the inner layer of the arteries.^1^ This process begins with the infiltration of low-density lipoprotein (LDL) particles into the vascular wall, where they undergo oxidation.^2^ Oxidized LDL is perceived as a foreign substance by the vascular endothelium, triggering an immune response and leading to the migration of inflammatory cells to the site.^3^ As this inflammatory response persists, atherosclerotic plaques form, narrowing the vascular lumen and impairing blood flow. Plaque rupture can result in thrombosis, leading to severe clinical outcomes such as myocardial infarction or stroke.^1^

It is now recognized that atherosclerosis is not solely a result of lipid accumulation but also an inflammatory disease.^3^ This paradigm shift in understanding has led to an increased focus on targeting inflammatory pathways as a complementary approach to lipid-lowering therapies. The oxidation of LDL by endothelial cells induces the migration of inflammatory cells into the vascular wall, promoting the differentiation of monocytes into macrophages.^2^ Macrophages phagocytose oxidized LDL, transforming into foam cells that secrete inflammatory cytokines, including interleukin-1 beta (IL-1β), interleukin-6 (IL-6), and tumor necrosis factor-alpha (TNF-α).^3^ These three cytokines were selected for focused analysis in this narrative review because they play central and well-characterized roles in the initiation and progression of atherosclerosis. IL-1β and IL-6 are key drivers of endothelial dysfunction and plaque destabilization, while TNF-α is involved in amplifying the inflammatory cascade and enhancing cellular adhesion, making them particularly relevant therapeutic targets.

The activation of these cytokines perpetuates inflammation and contributes to the progression of atherosclerotic plaques. IL-1β and IL-6 play significant roles in inflammatory signal transduction, leading to endothelial dysfunction and plaque progression.^4^ Meanwhile, TNF-α enhances cellular adhesion, facilitating the migration of inflammatory cells to plaques and compromising plaque stability, thereby increasing the risk of thrombosis.^5^

Biological agents targeting the inflammatory components of atherosclerosis aim to mitigate disease progression by blocking specific inflammatory pathways.^6^ Canakinumab, an IL-1β inhibitor, interrupts the inflammatory cascade and may enhance plaque stability.^4^ Tocilizumab, which blocks the IL-6 receptor, reduces inflammatory processes and improves endothelial function.^6^ TNF-α inhibitors (eg, adalimumab, infliximab) suppress vascular wall inflammation by reducing monocyte and macrophage activation.^1^

Studies evaluating the effects of these biological agents on atherosclerosis have demonstrated their potential to slow disease progression and reduce the risk of cardiovascular events.^7^ The CANTOS (Canakinumab Anti-Inflammatory Thrombosis Outcomes Study) trial showed that IL-1β inhibition reduces inflammation and lowers the incidence of recurrent cardiovascular events in patients with a history of myocardial infarction.^8^ This highlights the pivotal role of inflammation in cardiovascular risk management and supports the integration of anti-inflammatory strategies into standard treatment algorithms. Observational studies on patients with autoimmune diseases have reported that TNF-α inhibitors can slow the progression of atherosclerotic plaques and improve vascular function by reducing the inflammatory burden.^9^ IL-6 receptor inhibitors, such as tocilizumab, have also been found to decrease vascular stiffness and alleviate endothelial dysfunction by reducing plaque inflammation.^10^ However, further clinical trials are needed to understand the long-term effects of these agents.

The objective of this narrative review is to provide a comprehensive analysis of the potential role of anti-inflammatory biological agents in the management of atherosclerosis. Specifically, it will address the following questions: Which patient populations may benefit most from these therapies? How can these agents synergize with statins and other cardiovascular treatments? What are the long-term clinical benefits and risks associated with these drugs? Which emerging biological agents may offer improved efficacy in the future?

In conclusion, considering the central role of inflammation in atherosclerosis, anti-inflammatory biological agents represent a novel therapeutic approach for disease management. However, further evidence is necessary to support their routine clinical application. Future studies focusing on long-term outcomes and comparative effectiveness with standard therapies will be essential to establish their definitive role in clinical practice.

## Review

### Materials and Methods

#### Overview

This study is a narrative review that provides a comprehensive examination of the current literature on how biological therapies can modulate inflammation in atherosclerosis. Narrative reviews aim to synthesize existing evidence to provide a broad perspective on a specific research question. In this case, the study focuses on the inflammatory components of atherosclerosis and the potential therapeutic effects of biological agents.

#### Eligibility Criteria

The studies included in this narrative review were selected based on their relevance to the relationship between atherosclerosis and inflammation, with particular attention to the effects of biological therapies targeting inflammatory cytokines. Specifically, research on the inhibition of interleukin-1 beta (IL-1β), interleukin-6 (IL-6), and tumor necrosis factor-alpha (TNF-α) was prioritized. Human studies, including observational studies, clinical trials, and randomized controlled trials (RCTs), were included.

The inclusion criteria comprised peer-reviewed articles that focused on inflammatory mechanisms in atherosclerosis, the application of biological agents in cardiovascular disease management, and relevant clinical or biochemical outcomes. Studies evaluating the effects of biological therapies on inflammatory markers and atherosclerotic plaque progression were also included.

Exclusion criteria involved non-cardiovascular studies, case reports, editorials, conference abstracts, and studies with inadequate methodology or insufficient data. Articles focusing solely on animal models or in vitro experiments were also excluded to ensure the relevance of findings to human clinical practice.

#### Information Sources

Data for this narrative review were obtained from reliable academic databases, including PubMed, Scopus, and Google Scholar. The literature search primarily focused on articles published within the last 10 years, with particular emphasis on research from the past 5 years to ensure the inclusion of the most recent findings. However, landmark studies and pivotal trials contributing to the understanding of inflammation and biological therapies in atherosclerosis were also included, even if published earlier.

Key search terms used included “atherosclerosis”, “inflammation”, “biological therapy”, “IL-1β inhibitors”, “IL-6 inhibitors”, and “TNF-α inhibitors”. Large-scale clinical trials such as the CANTOS (Canakinumab Anti-Inflammatory Thrombosis Outcomes Study) were specifically reviewed. Full-text articles were accessed for comprehensive analysis.

Recent emphasis was placed on biological agents investigated in contemporary cardiovascular trials, including anakinra, a recombinant IL-1 receptor antagonist evaluated in the VCUART3 study; tocilizumab, an IL-6 receptor blocker assessed in the ASSAIL-MI and NSTEMI trials; and rituximab, a CD20+ B-cell depleting monoclonal antibody examined in the RITA-MI trial.

Secukinumab, an IL-17A inhibitor, was also included due to its potential cardiovascular benefits demonstrated through improved endothelial function.

In addition, novel lipid-lowering strategies such as PCSK9 inhibitors—particularly alirocumab—have shown promise in further reducing cardiovascular risk when used in conjunction with standard high-intensity statin therapy.

#### Selection Process

The selection process was carried out in two stages. In the first stage, titles and abstracts of the retrieved articles were screened to identify those directly related to the interplay between atherosclerosis, inflammation, and biological therapies. Studies that met the inclusion criteria underwent a full-text review to assess their methodological quality, sample size, study design, follow-up duration, and statistical analyses.

Priority was given to randomized controlled trials (RCTs), prospective and retrospective cohort studies, case-control studies, and systematic reviews or meta-analyses. Articles were selected based on their contribution to understanding the effects of biological therapies on inflammation, atherosclerotic plaque stability, and cardiovascular event reduction.

Independent assessments were performed to ensure the reliability and validity of the selection process. Studies with clear methodologies, robust statistical analyses, and clinically relevant outcomes were prioritized for inclusion.

#### Data Extraction

Data extraction was conducted systematically, focusing on key variables from each study. Information on study design, sample size, population characteristics, type of biological therapy used, dosage, and treatment duration was collected. Additionally, data on inflammatory biomarkers such as C-reactive protein (CRP), IL-1β, IL-6, and TNF-α were recorded.

Clinical outcomes, including the incidence of cardiovascular events, major adverse cardiovascular events (MACE), mortality rates, and hospitalization durations, were also extracted. Adverse events related to biological therapies, including infection risk and immune suppression, were documented to evaluate the safety profile of the treatments.

Comparative data between treatment groups and control or placebo groups were analyzed to determine the efficacy of biological therapies in reducing inflammation and improving cardiovascular outcomes. Statistical methodologies used in the studies, such as hazard ratios, confidence intervals, and p-values, were carefully noted to assess the robustness of the findings.

Overall, this comprehensive data extraction approach ensures an in-depth understanding of the role of biological therapies in the management of atherosclerosis. The findings are synthesized to provide a balanced perspective on the clinical utility, safety, and limitations of these therapeutic strategies.

## Results

### What Is Atherosclerosis?

Atherosclerosis is a progressive vascular disease characterized by chronic inflammation and lipid accumulation in the arterial walls. This process begins with endothelial cell dysfunction and eventually leads to the formation of atherosclerotic plaques. Damage to endothelial cells facilitates the migration of inflammatory cells and lipid particles into the vascular wall. When low-density lipoprotein (LDL) particles penetrate the subendothelial region, they undergo oxidation, increasing oxidative stress. This triggers the adhesion of monocytes to the endothelial surface and their differentiation into macrophages.^11^

Macrophages phagocytose oxidized LDL, transforming into foam cells that form the primary components of atherosclerotic plaques. Over time, these plaques become enriched with fibrotic tissue and necrotic cores. The rupture of atherosclerotic plaques can lead to thrombosis, causing severe cardiovascular events such as myocardial infarction or ischemic stroke.^12^

The progression of atherosclerosis is not solely limited to lipid accumulation; the inflammatory process also significantly impacts plaque stability. While stable plaques are characterized by a thick fibrous cap, plaques with thin fibrous caps and large necrotic cores carry a higher risk of rupture. Therefore, inflammation is considered a pivotal factor in every stage of the atherosclerotic process.^13^

### Relationship Between Atherosclerosis and Inflammation

Atherosclerosis is a process driven by chronic inflammation. Oxidized LDL particles initiate the inflammatory response by promoting endothelial dysfunction. Endothelial cells release adhesion molecules, such as vascular cell adhesion molecule-1 (VCAM-1) and intercellular adhesion molecule-1 (ICAM-1), along with chemokines that facilitate monocyte adhesion to the vascular wall. After migrating into the vascular wall, monocytes differentiate into macrophages and secrete inflammatory cytokines.^14^

**Figure 1:**
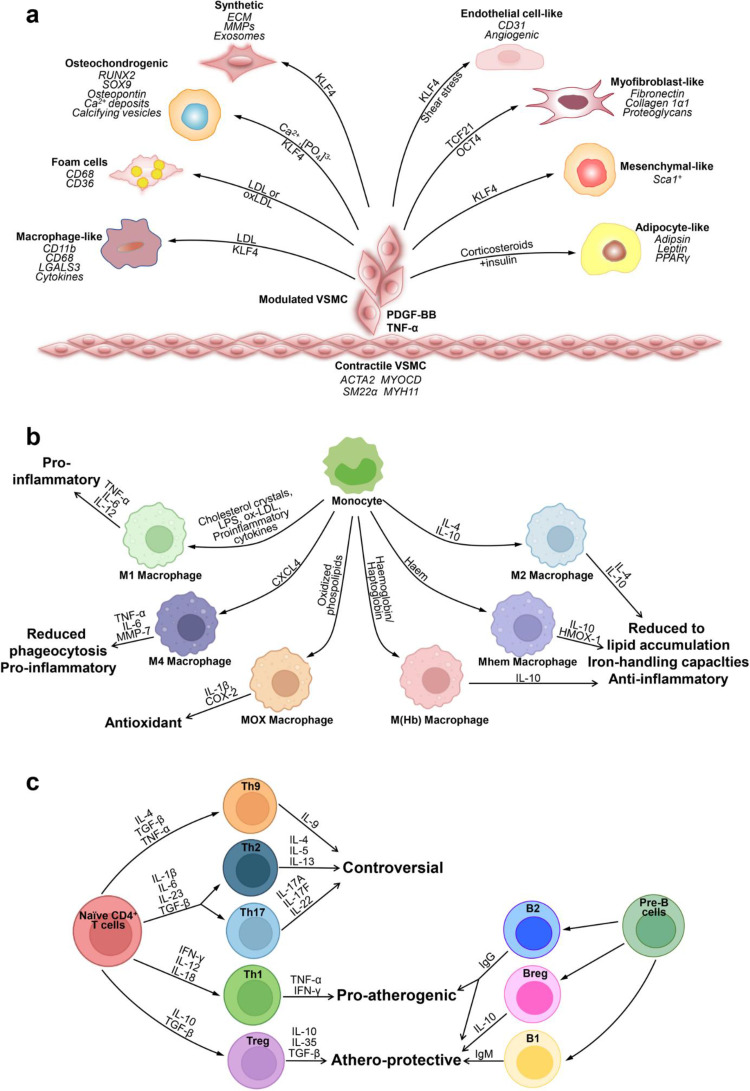
Key cells involved in atherosclerosis. (**a**) Phenotypic switching of VSMCs in atherosclerosis. In the healthy arterial wall, VSMCs are a contractile phenotype expressing contractile proteins (ACTA2, SM22α, Myocardin (MYOCD), and MYH11). Upon PDGF-BB and TNF-α, VSMCs switch to a synthetic phenotype, which increases the production of ECM, exosomes, pro-inflammatory cytokines, and MMPs. VSMCs release calcifying vesicles to propagate vascular calcification. KLF4 promotes phenotypic switching of VSMCs into foam-like, macrophage-like, osteochondrocyte-like, adipocyte-like, and Sca1+ mesenchymal-like VSMCs. Shear stress induces transdifferentiation of VSMCs into endothelial-like cells. The transcription factors TCF21 and OCT4 (octamer binding transcription factor) promote modulation of VSMCs into atheroprotective myofibroblast-like phenotype. (**b**) Plasticity and function of macrophages in atherosclerosis. Monocytes differentiate toward various phenotypes of macrophages in response to stimuli in atherosclerotic lesions. Among them, M1 macrophages secret pro-inflammatory cytokines; M2, M (Hb), and Mhem phenotypes are anti-inflammatory; Mox macrophages exhibit an antioxidant effect; and M4 phenotypes express pro-inflammatory cytokines and have impaired phagocytosis. (**c**) Lymphocytes in atherosclerosis. CD4+ T cells can differentiate into distinct lineages including T helper 1 (Th1), Th2, Th17, T regulatory (Treg), and many other Th cells. Th1 cells produce TNF-α and IFN-γ, indicating a pro-inflammatory and pro-atherogenic role of Th1 cells. Treg cells promote inflammatory resolution and dampen atherosclerosis progression via the production of IL-10 and TGFβ. The effect of Th2, Th9, and Th17 cells on the development of atherosclerosis remains controversial. B cells can exert both a pathogenic and protective role in atherosclerosis. B cells have two main subsets B1 and B2 cells. B1 cells exert an atheroprotective effect by the release of IgM antibodies against oxidation-specific epitopes. Similarly, Breg cells also act atheroprotective by the production of IL-10. B2 cells exhibit both pro-atherogenic and atheroprotective depending on the inflammatory microenvironment. Adapted from Kong P, Cui Z-Y, Huang X-F, et al. Inflammation and atherosclerosis: signaling pathways and therapeutic intervention. *Signal Transduc Targeted Therapy*. 2022;7(1):131. Creative Commons.^15^

Key cytokines involved in this process include tumor necrosis factor-alpha (TNF-α), interleukin-1 beta (IL-1β), and interleukin-6 (IL-6). Figure 1 illustrates the key inflammatory pathways in atherosclerosis, highlighting the roles of cytokines such as IL-1β, IL-6, and TNF-α in endothelial dysfunction and plaque progression (Figure 1).

TNF-α promotes the recruitment of inflammatory cells and activates endothelial cells, while IL-1β and IL-6 sustain the inflammatory response. Activated macrophages and T cells contribute to foam cell formation and plaque growth.^16^

Oxidative stress further amplifies the inflammatory process. Oxidized LDL increases the production of reactive oxygen species (ROS), exacerbating endothelial cell damage. This leads to a heightened inflammatory response and the destabilization of atherosclerotic plaques. Vascular remodeling induced by inflammation results in arterial stiffening and luminal narrowing.^17^

Elevated levels of inflammatory markers, such as C-reactive protein (CRP) and IL-6, have been identified as predictors of atherosclerotic cardiovascular disease in clinical studies. Consequently, targeting inflammation is considered a promising strategy to prevent or slow the progression of atherosclerosis.^18^

### Inflammation and Biological Agents

Biological agents are a class of drugs produced by cultivating bacterial, yeast, plant, or animal cells in large-scale cell cultures, followed by purification processes. Unlike conventional small-molecule drugs that are chemically synthesized or extracted from plants, biologics are typically purified proteins derived from living systems or human blood. They are structurally complex and designed to target specific molecular pathways. While many biologics are used to suppress inflammation, particularly in autoimmune and cardiovascular conditions, their applications also extend to oncology, hematology, and metabolic diseases. In the context of atherosclerosis, certain biologic agents have shown potential in modulating key components of the inflammatory response. (Table 1).

**Table 1:** Biological Agents and Targeted Pathways

Biological Agent	Targeted Pathway	Primary Use	References
Canakinumab	IL-1β Inhibition	Rheumatoid Arthritis, Cardiovascular Disease	[4,19]
Anakinra	IL-1 Receptor Antagonism	STEMI, Inflammatory Diseases	[20]
Tocilizumab	IL-6 Receptor Blockade	RA, Giant Cell Arteritis, STEMI, NSTEMI	[21,22]
Rituximab	CD20+ B-cell Depletion	Autoimmune Diseases, STEMI	[23]
Secukinumab	IL-17A Inhibition	Psoriasis, Endothelial Function	[24]
Alirocumab	PCSK9 Inhibition	Hyperlipidemia, STEMI	[25]
Adalimumab	TNF-α Inhibition	Rheumatoid Arthritis, Psoriatic Arthritis	[15,22]
Infliximab	TNF-α Inhibition	Rheumatoid Arthritis, Inflammatory Bowel Disease	[22]
Etanercept	TNF-α Inhibition	Ankylosing Spondylitis, Psoriatic Arthritis	[22]

These include monoclonal antibodies, receptor antagonists, and cytokine inhibitors, which are widely used in the treatment of chronic inflammatory diseases. By targeting key inflammatory cytokines such as IL-1β, IL-6, and TNF-α, these agents exert therapeutic effects.^26^

IL-1β inhibitors play a critical role in the early stages of inflammation. IL-1β is a central mediator in initiating and sustaining the inflammatory response. Canakinumab, a monoclonal antibody targeting IL-1β, prevents excessive activation of the inflammatory cascade.^19^ It has demonstrated efficacy in treating conditions such as rheumatoid arthritis, systemic juvenile idiopathic arthritis, and cryopyrin-associated periodic syndromes.^19^ Moreover, in the CANTOS trial, canakinumab significantly reduced the incidence of recurrent cardiovascular events in post-myocardial infarction patients with elevated hs-CRP, confirming the role of IL-1β inhibition in cardiovascular protection.^27^ Additionally, canakinumab has shown promising results in inflammation-driven diseases, including cardiovascular diseases.^21^

IL-6 inhibitors also suppress the inflammatory response by blocking the actions of IL-6, a cytokine that sustains inflammation. Tocilizumab, an IL-6 receptor antagonist, prevents IL-6 from exerting its pro-inflammatory effects. The ASSAIL-MI trial demonstrated that tocilizumab significantly increased myocardial salvage in STEMI patients and reduced microvascular obstruction, suggesting a protective role in ischemia-reperfusion injury.^22^ Similarly, in patients with NSTEMI, tocilizumab reduced CRP and troponin T release, indicating attenuation of both systemic inflammation and myocardial injury.^28^ However, it should be noted that IL-6 blockade with tocilizumab has been associated with transient increases in total cholesterol, LDL, and triglyceride levels, which may necessitate close lipid monitoring during therapy.^28^ It is commonly used in the management of rheumatoid arthritis and giant cell arteritis.^29^ IL-6 inhibition effectively reduces systemic inflammation and prevents inflammation-related tissue damage.^27^

TNF-α inhibitors target TNF-α’s pivotal role in initiating the inflammatory response. Agents such as infliximab, adalimumab, and etanercept are widely used to manage inflammatory diseases by blocking TNF-α activity.^30^ These inhibitors have demonstrated efficacy in controlling symptoms and preventing disease progression in conditions like rheumatoid arthritis, ankylosing spondylitis, psoriatic arthritis, and inflammatory bowel disease.^16^

While biological agents offer a more targeted and effective therapeutic approach by modulating specific inflammatory pathways, their immunosuppressive effects can increase the risk of infections and malignancies.^23^,^31^ Therefore, their use is generally reserved for patients who do not respond to standard therapies or those with severe inflammatory diseases. Long-term studies are essential to further establish their efficacy and safety profiles in clinical practice.

Beyond cytokine inhibitors, recent attention has turned to B-cell modulation as a novel anti-inflammatory approach in cardiovascular disease. In the RITA-MI trial, a single dose of rituximab, a monoclonal antibody targeting CD20+ B cells, was found to be safe and led to substantial B-cell depletion in acute STEMI patients without adverse immunosuppressive effects, laying the foundation for future trials exploring its therapeutic potential.^24^

Secukinumab, a fully human monoclonal antibody that inhibits IL-17A, has shown improvement in vascular endothelial function in psoriasis patients, as demonstrated in the CARIMA study. Although conducted in a non-cardiac population, these findings suggest that IL-17A inhibition may have favorable effects on cardiovascular risk profiles through endothelial modulation.^25^

In addition, alirocumab, a PCSK9 inhibitor, has been associated with early and substantial LDL-C reduction in STEMI patients undergoing PCI, as shown in the EPIC-STEMI trial. Its anti-inflammatory and lipid-lowering dual action may support long-term vascular protection when combined with cytokine-targeted therapies.^20^

### Atherosclerosis and Biological Agents

The application of biological agents offers a promising therapeutic approach targeting the inflammatory nature of atherosclerosis. The CANTOS trial demonstrated that canakinumab, an IL-1β inhibitor, significantly reduced cardiovascular event risk by suppressing inflammation. Recent clinical trials have evaluated biological therapies agents for their potential to reduce cardiovascular events and inflammatory markers, offering comparative insights into inflammation-targeted strategies (Table 2). This study provided strong evidence supporting the treatability of inflammation in atherosclerosis.^21^

**Table 2:** Clinical Study Findings of Biological Anti-Inflammatory Therapies

Study	Agent Used	Primary Outcome	Key Finding	References
CANTOS	Canakinumab	15% Reduction in Cardiovascular Events	Reduced hs-CRP levels and recurrent events	[4,8]
VCUART3	Anakinra	Decreased Heart Failure Incidence	Reduced systemic inflammation and HF hospitalization	[20]
ASSAIL-MI	Tocilizumab	Increased Myocardial Salvage	Reduced microvascular obstruction in STEMI	[21]
NSTEMI-TCZ	Tocilizumab	Reduction in Troponin and CRP	Lower myocardial injury and systemic inflammation	[22]
RITA-MI	Rituximab	Safety and B-cell Depletion	>95% B-cell depletion; safe in STEMI	[23]
CARIMA	Secukinumab	Improved Endothelial Function	Increased FMD in psoriasis patients at high CV risk	[24]
EPIC-STEMI	Alirocumab	LDL-C Reduction Post-PCI	Early and effective lipid reduction in STEMI patients	[25]

In the VCUART3 trial, interleukin-1 receptor blockade with anakinra significantly reduced systemic inflammatory response in patients with ST-segment elevation myocardial infarction, resulting in a lower incidence of heart failure-related events.^32^

In addition, tocilizumab, an IL-6 receptor antagonist, has demonstrated beneficial effects in patients with acute coronary syndromes. In both the ASSAIL-MI and NSTEMI trials, tocilizumab was shown to reduce myocardial injury, systemic inflammation, and improve myocardial salvage, reinforcing its role in acute atherothrombotic events.^22^,^28^

TNF-α inhibitors have been associated with reduced cardiovascular risk, particularly in patients with inflammatory diseases. However, their direct effects on atherosclerosis remain unclear and require further investigation through long-term studies.^31^

Beyond cytokine blockade, B-cell modulation has emerged as a novel therapeutic approach. In the RITA-MI trial, rituximab, a monoclonal antibody targeting CD20+ B cells, achieved over 95% depletion of circulating B cells in STEMI patients without adverse safety signals, highlighting its potential in immunoinflammatory vascular disease.^24^

Moreover, the CARIMA study demonstrated that secukinumab, an IL-17A inhibitor, improved endothelial function in patients with psoriasis, suggesting vascular benefit through modulation of endothelial inflammation.^25^

**Figure 2:**
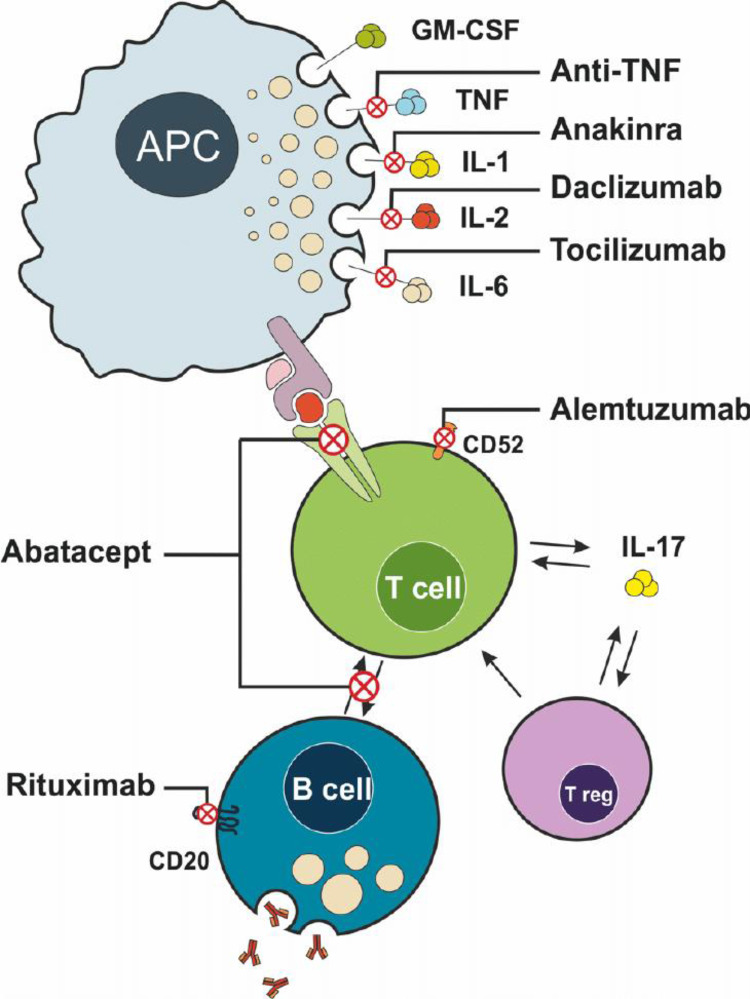
Mechanisms of action of commonly used biological agents targeting immune cells and cytokines in systemic inflammation. This illustration depicts the interaction between antigen-presenting cells (APCs), T cells, B cells, and regulatory T cells (Treg) in the immune response, and highlights the specific targets of several biological agents. APCs secrete multiple pro-inflammatory cytokines, including GM-CSF, TNF, IL-1, IL-2, and IL-6, which contribute to immune activation and inflammation. These cytokines are selectively inhibited by biological therapies: anti-TNF agents block TNF, anakinra inhibits IL-1, daclizumab targets IL-2, and tocilizumab blocks IL-6. The interaction between APCs and T cells is further modulated by Abatacept, which interferes with costimulatory signaling required for T cell activation. Alemtuzumab targets CD52 on T cells, leading to their depletion. Rituximab selectively depletes B cells by binding to CD20. Collectively, these agents modulate the immune response by inhibiting cytokine signaling, preventing cell activation, or depleting key immune cell populations. IL-17 production by T cells, as well as the regulatory feedback of Treg cells, is also illustrated as part of the inflammatory cascade. This figure is adapted from the work of Vela and Andrés titled “The Role of Biological Therapies in the Management of Systemic Vasculitis”, published in the book Advances in the Diagnosis and Treatment of Vasculitis by IntechOpen in 2011. Creative Commons.^35^

Alirocumab, a PCSK9 inhibitor, showed significant LDL-C reduction and early lipid stabilization in the EPIC-STEMI trial when administered during primary PCI, which may synergize with anti-inflammatory therapies in the management of atherosclerosis.^20^ PCSK9 inhibitors, such as alirocumab and evolocumab, are biological agents that not only provide potent lipid-lowering effects but also modulate inflammation through indirect mechanisms. By preventing the degradation of LDL receptors, they significantly reduce circulating LDL cholesterol and contribute to plaque stabilization. Furthermore, evidence suggests that early administration of PCSK9 inhibitors in acute coronary syndrome patients can lead to rapid lipid control and attenuation of systemic inflammation. These effects may improve endothelial function and reduce atherosclerotic progression. Due to these benefits, PCSK9 inhibitors are now recommended in international cardiovascular guidelines for high-risk patients who fail to achieve LDL-C targets despite statin therapy.^20^,^33^,^34^ Figure 2 illustrates the specific molecular targets of biologic agents discussed in this section, including IL-1β, IL-6, TNF-α, IL-17A, CD20+ B cells, and PCSK9. The schematic highlights how each therapy interrupts inflammatory signaling pathways involved in atherogenesis (Figure 2).

In conclusion, biological agents present a promising therapeutic option for reducing inflammation and mitigating its effects on atherosclerosis. Future large-scale and long-term studies will be crucial in determining the efficacy and safety of these therapies in routine clinical practice.

## Discussion

Atherosclerosis remains the most common cause of cardiovascular diseases (CVD) worldwide and has long been considered a lipid accumulation-based disease. However, the critical role of inflammation in the pathogenesis of atherosclerosis is now widely acknowledged.^11^ Inflammation plays a pivotal role in every stage of atherosclerosis, from its initiation to its progression, ultimately leading to cardiovascular events. Therefore, biologic agents targeting inflammation have emerged as promising therapeutic options for atherosclerotic diseases. Recent large-scale studies such as CANTOS have provided significant insights into the effectiveness and limitations of inflammation-targeted therapies.^4^,^25^ This article explores the role of inflammation in atherosclerosis and highlights clinical trial findings related to biologic agents used in this context.

Atherosclerosis is a chronic disease characterized by the combination of lipid accumulation and inflammatory processes within the arterial wall. The infiltration and oxidation of low-density lipoprotein (LDL) particles into the vascular wall initiate the atherosclerotic process. Oxidized LDL (ox-LDL) triggers an inflammatory response in endothelial cells, leading to the increased expression of vascular cell adhesion molecule-1 (VCAM-1) and intercellular adhesion molecule-1 (ICAM-1). These molecules facilitate the attachment and migration of monocytes to the endothelium.^11^

Upon entering the intima layer, monocytes differentiate into macrophages and engulf oxidized LDL, forming foam cells. Foam cells release pro-inflammatory cytokines and chemokines, further amplifying inflammation. Key cytokines such as tumor necrosis factor-alpha (TNF-α), interleukin-1 beta (IL-1β), and interleukin-6 (IL-6) play essential roles in this process.^16^ Chronic inflammation leads to the progression of atherosclerotic plaques, thinning of the fibrous cap, and an increased risk of plaque rupture. Plaque rupture may result in thrombus formation, leading to major cardiovascular events such as myocardial infarction, stroke, and sudden cardiac death.^11^

Therefore, targeting the inflammatory effects in atherosclerosis presents significant potential for the prevention and treatment of cardiovascular diseases.

The CANTOS study is one of the most comprehensive and influential trials directly targeting inflammation in the pathogenesis of atherosclerosis. Canakinumab, an IL-1β inhibitor, specifically blocks IL-1β, a central mediator of the inflammatory response, thereby suppressing inflammation. The study included patients with a history of myocardial infarction and a high-sensitivity C-reactive protein (hs-CRP) level of ≥2 mg/L, focusing on a population with elevated inflammatory markers.^4^,^8^,^19^ According to the CANTOS study results, patients treated with canakinumab experienced a 15% reduction in cardiovascular events compared to the placebo group.^8^ Additionally, a significant decrease in hs-CRP levels was observed, with a strong correlation between reduced hs-CRP levels and decreased cardiovascular events. These findings demonstrate that inflammation in atherosclerosis can be directly targeted and that inflammation-focused therapies can effectively reduce cardiovascular risk.^1^ However, the absence of a significant improvement in overall mortality rates with canakinumab treatment suggests that suppressing inflammation alone may not be sufficient.^4^ Furthermore, factors such as the increased risk of infections and the high cost of treatment limit the widespread application of this therapy.^23^ The CANTOS study results indicate that targeting inflammation may be particularly beneficial in patients with elevated inflammatory markers, emphasizing the importance of individualized treatment decisions.

Supporting the IL-1 pathway hypothesis, the VCUART3 trial demonstrated that anakinra, a recombinant IL-1 receptor antagonist, significantly reduced systemic inflammatory response and lowered the incidence of new-onset heart failure and hospitalization for heart failure in STEMI patients.^32^

Interleukin-6 (IL-6) is another key inflammatory mediator, and its blockade has shown promise in cardiovascular settings. In the ASSAIL-MI trial, tocilizumab—a monoclonal antibody targeting IL-6 receptors—was shown to increase myocardial salvage and reduce microvascular obstruction in patients with acute STEMI.^22^ Similarly, in a double-blind trial in NSTEMI patients, a single dose of tocilizumab led to a significant reduction in CRP and troponin T levels, indicating an attenuated inflammatory and myocardial injury response.^28^

Expanding the scope of inflammatory modulation, the RITA-MI trial assessed the safety and immunologic impact of rituximab, a CD20+ B-cell depleting monoclonal antibody, in STEMI patients. The trial found rituximab to be safe and effective in achieving rapid and profound B-cell depletion, establishing feasibility for adaptive immune-targeted therapies in acute coronary syndromes.^24^

Beyond cytokines and B cells, the CARIMA trial investigated secukinumab, an IL-17A inhibitor, in psoriasis patients. Over 52 weeks, secukinumab significantly improved endothelial function as measured by flow-mediated dilation (FMD), supporting the hypothesis that IL-17A inhibition may confer cardiovascular benefit through endothelial stabilization and vascular protection.^25^

Additionally, lipid-lowering therapies with anti-inflammatory properties are being explored for their additive value. The EPIC-STEMI trial assessed alirocumab, a PCSK9 inhibitor, in patients undergoing primary PCI for STEMI. The study found that early administration of alirocumab achieved a significantly greater reduction in LDL-C, which may contribute to plaque stabilization and enhanced recovery.^20^

These findings collectively support the evolving paradigm that inflammation is not just a contributor but a therapeutic target in atherosclerosis. From cytokine blockade to B-cell modulation and vascular immune stabilization, biologic agents represent a rapidly expanding frontier. Future approaches will require integration of biomarkers, patient stratification, and long-term outcome studies to guide optimal and personalized inflammation-targeted cardiovascular care.

## Limitations

Although the use of biologic agents targeting inflammation in the treatment of atherosclerosis is promising, several limitations exist. These therapies are generally effective in patients with high levels of inflammatory markers, while their efficacy in individuals with low inflammation levels remains uncertain. Additionally, the lack of long-term safety data and the increased risk of infections pose significant challenges during treatment. High costs and limited accessibility are also factors that restrict the widespread use of biologic agents.

Furthermore, as inflammation varies on an individual level, the response to therapy can differ. The development of personalized treatment approaches and the use of biomarkers may facilitate appropriate patient selection. Moreover, long-term studies are necessary to clarify the effects of existing biologic agents on cardiovascular mortality. In conclusion, considering the limitations of biologic agents, the development of more comprehensive and personalized treatment strategies could provide significant progress in the management of atherosclerosis.

## Conclusions

Atherosclerosis is a multifactorial and chronic inflammatory disease that remains a leading cause of cardiovascular morbidity and mortality worldwide. The recognition of inflammation as a central driver of plaque formation and instability has led to the emergence of biologic agents that selectively target key inflammatory pathways. Among these, agents inhibiting IL-1β, IL-6, IL-17A, and CD20+ B cells have demonstrated encouraging results in both systemic inflammatory diseases and cardiovascular conditions. These therapies offer the potential not only to reduce inflammation but also to stabilize atherosclerotic plaques and prevent acute vascular events.

Despite their promising effects, the clinical use of biologic agents is currently limited by challenges such as high cost, immunosuppressive risks, and the need for precise patient selection. Their efficacy appears to be most pronounced in individuals with elevated inflammatory markers, highlighting the importance of personalized treatment strategies. The integration of biomarkers into clinical decision-making may improve treatment outcomes by identifying patients who would benefit most from these therapies.

In conclusion, biologic anti-inflammatory agents represent an evolving and targeted approach in the management of atherosclerosis. As evidence continues to accumulate, future research should prioritize long-term safety data, cost-effectiveness analyses, and real-world applicability. These efforts will be critical in determining how these therapies can be more broadly and effectively implemented in cardiovascular medicine.

## Abbreviations

IL-1β, Interleukin-1 beta; IL-6, Interleukin-6; TNF-α, Tumor Necrosis Factor-alpha; CANTOS, Canakinumab Anti-Inflammatory Thrombosis Outcomes Study; CIRT, Cardiovascular Inflammation Reduction Trial.
